# Plasma Bioscience and Medicine Molecular Research

**DOI:** 10.3390/ijms24119174

**Published:** 2023-05-24

**Authors:** Nagendra Kumar Kaushik, Eun Ha Choi

**Affiliations:** Plasma Bioscience Research Center, Department of Electrical and Biological Physics, Kwangwoon University, Seoul 01897, Republic of Korea

## 1. Introduction

This special issue delivers an applied and basic platform for exchanging advanced approaches or research performance that link the plasma physics research in cell biology, cancer treatments, immunomodulation, stem cell differentiation, nanomaterial synthesis, and their applications, agriculture and food processing, microbial inactivation, water decontamination, and sterilization applications, including in vitro and in vivo research. Plasma is recently used in many ways in the field of agriculture and food processing. Although the processes that accompany the development of plasma-based agriculture or biological applications are known, the underlying molecular basis is less understood. For that, knowing new mechanisms and molecular events that participate in this plasma-induced biological process is fundamental to understanding its development and would allow having new targets for biological applications or new treatment strategies. Several manuscripts were submitted to this Special Issue, of which 13 finest manuscripts were accepted for publication. Various topics have been addressed in this Special Issue, specially plasma-activated water (one article), plasma-induced cell motility and wound healing (two articles), plasma-based inactivation of environmentally transmitted pathogens or biofilms (three articles), cancer treatment application (three articles), plasma-based biological production using beneficial microbes (one article), plasma-based transport and modification of via lipid bilayer (one article), plasma-based smart and green agriculture (one article) and plasma-based stem cell differentiation (one article). We are obliged to all these hardworking researchers who authored these impressive articles, and we are delighted to compile all the high-quality articles for this platform. The following sections mentioned below specify a summary of each of the articles published in this Special Issue.

## 2. Contributions in Various Fields

### 2.1. Plasma-Generated Species in Water

Nonthermal plasma (NTP) is increasingly being used in various fields, including energy, environmental, and biological and material treatment applications. One such application is the generation of plasma-treated water (PTW) also known as plasma-activated water (PAW) or plasma-stimulated water (PSW), which can have several active species inside for biological or material treatment applications. The interaction of plasma with water is a complex process that generates high reactivity and UV radiation. PTW has been shown to enhance the stress tolerance, germination, and growth rate of plants, making it a promising solution for agricultural problems. Razzokov et al. used molecular dynamics (MD) simulations to investigate the permeation of plasma-generated species from the vacuum into the water bulk to understand the mechanism of transportation [1]. The study found that hydrophilic reactive oxygen and nitrogen species i. e. RONS (OH, HO_2_, H_2_O_2_, trans-HNO_2_, cis-HNO_2_, HNO_3_, ONOOH, and N_2_O_4_) could enter the vacuum-water interface and the water bulk phase, while the translocation probabilities of other RONS (O_3_, NO, and NO_2_) into the water bulk were comparatively less favorable due to free energy barriers. The study provides a basis for the design of experiments for PTW production with controlled RONS composition, serving as a starting point for other RONS that still lack experimental data. The study is significant in developing specific types of RONS-enriched PTW that can be applied in plasma medicine and agriculture fields.

### 2.2. Plasma-Induced Cell Motility and Wound Healing 

Generally human or animal cells elongate perpendicularly to the electric field and move in the direction of the electric field. Recently, Tada et al., showed that even plasma-based nanosecond pulsed current elongates cells, however, the direction of the movement and elongation was not defined [2]. In this research article, they used observed cell physiological behaviors after applying plasma stimulated pulse current. Their results showed that nanosecond pulsed currents elongate cells but do not affect the movement direction. It is also reported that the behavior of the cell is based on the plasma-based nanosecond current applied. This research work paper gives direction toward novel insight related to plasma-based electrical aspects.

NTP is known to have a positive effect on sterilization and the wound-healing process. Marches et al. demonstrated the effect of a direct helium plasma jet treatment on skin cells such as immortalized and primary keratinocytes obtained from abdominal skin samples [3]. In this study, cells were exposed to helium jet plasma for 10–120 s. In this study, they found the generation of hydrogen peroxide, nitrite, and nitrate in micromolar concentration. It is shown that long plasma exposures (≥60 s) were toxic, ameliorates skin cell migration, enhanced heat shock protein 27 (HSP27) expression, and induced oxidative stress inside the cells. However, short plasma exposure (<60 s) was not toxic, and did not affect skin cells’ physiological phenomena, but they did fasten wound closure via keratinocyte migration. It is concluded that helium-plasma treatments can improve wound healing by activating migration factors. This research works to justify the use of plasma for wound healing purposes. Further cellular and molecular mechanism is warranted for future directions in the wound healing processes. 

### 2.3. Effect on Environmentally Transmitted Pathogens and Biofilms 

The emergence of antibiotic-resistant bacteria is a major concern, and there is an urgent need for effective and biocompatible therapeutic strategies against these bacteria. Borkar et al. investigated the inactivation efficiency and mechanisms of plasma-generated nitric oxide water (PG-NOW) on pathogenic water, air, soil, and foodborne Gram-negative and Gram-positive bacteria [4]. The results showed that PG-NOW treatment effectively inhibited the growth of bacteria and caused intracellular nitric oxide (NO) accumulation. The study also evaluated the expression of genes associated with nitrosative and oxidative stress in bacterial cells after PG-NOW treatment. The soxRS expression was enhanced, whereas the oxyR expression was decreased following PG-NOW treatment. Additionally, the disruption of cell morphology was observed using scanning electron microscopy (SEM) analysis. The study concluded that PG-NOW could be a promising antibacterial treatment system without causing resistance in bacteria, and it could be administered through various delivery methods for the treatment of bacterial infections. Future research should focus on discovering the pharmacokinetic parameters of PG-NOW treatment technologies for their safe administration. Overall, the study provides evidence of an initiation point for further development of PG-NOW-based antibacterial treatments. 

Liu et al. investigated the effectiveness of humidified air dielectric barrier discharge (DBD) plasma in inactivating Escherichia coli, Staphylococcus aureus, and bacteriophages in biofilms containing DNA, NaCl, carbohydrates, and proteins [5]. The research found that the inactivation efficiency of the microbes was dependent on the thickness and constituents of the biofilms, and plasma treatment time. However, proteins in the biofilm were highly resistant to the inactivation of microbes in biofilms due to the existence of unstable functional groups. The study concluded that UV irradiation was not responsible for the effective inactivation of microbes, and short-lived active species generated in the humidified air plasma were not directly involved in the inactivation process. Instead, they reacted with other species to generate long-lived species, which diffused into the biofilms to generate short-lived species, such as ONOOH and OH. The geminated NO_2_ and OH pair formed due to the homolysis of ONOOH caused the synergistic oxidation of various organic molecules in the aqueous solution. The unsaturated fatty acids, cysteine-rich proteins, and sulfur-methyl thioether groups in the proteins were easily oxidized by the geminated NO_2_ and OH pair, leading to a decrease in the density of ONOOH in the biofilms. This study suggests that the humidified air DBD plasma can efficiently inactivate microbes in biofilms containing DNA, NaCl, carbohydrates, and proteins, and the long-lived R species may play a critical role in this process.

PTW has been shown to have antifungal effects on various fungal pathogens, but its effect on the newly emerged mushroom pathogen Cryptococcus pseudolongus is unknown. Lee et al. investigated the capture of nitrate by magnesium ions in PTW and its antifungal effect on C. pseudolongus [6]. The plasma jet generated several reactive oxygen and nitrogen species, and plasma treatment of deionized water produced acidic PTW. However, when plasma was injected into magnesium water, the resulting plasma-activated magnesium water (PA-Mg-W) tended to be neutralized due to the reduction in plasma-generated hydrogen ions by electrons released from the zero-valent magnesium. Optical absorption and Raman spectra confirmed that nitrate ions were the dominant reactive species in both PTW and PA-Mg-W. The free nitrate content was controlled to be lower in the PA-Mg-W than in the PAW due to the formation of nitrate salts by magnesium ions. The antifungal effects of PTW and PA-Mg-W were tested on C. pseudolongus, with both having a significant effect on cell viability. However, the effectiveness of PA-Mg-W was higher than that of PTW, with cell viability being higher in the former. This study demonstrates that the antifungal effect of PTW can be manipulated using nitrate capture, and the use of PTW for problematic fungus control is promising. The study also extends the application of PTW from Ascomycota fungi to Basidiomycota fungi and suggests its potential use in various antifungal therapies.

### 2.4. Cancer Treatment Application 

Melanoma is an aggressive and malignant cancer that often relapses and metastasizes to various organs. NTP, a novel anticancer tool that utilizes abundant RONS, has been studied for its differential effects on non-cancerous and cancer cells. In vitro, experiments using melanoma and non-cancerous skin fibroblast cells showed that the production of intracellular RONS increased remarkably only in melanoma cancer cells after exposure to NTP, while the non-cancerous cells seemed to have a higher tolerance for RONS [7]. Moreover, cancer cells morphed from spread to round cell shapes after plasma exposure, suggesting that they were more affected than non-cancerous cells in the same plasma condition. The results suggest that differential sensitivities of non-cancerous skin and melanoma cells to NTP-induced RONS can enable the applicability of NTP in anticancer therapy. The selectivity of NTP treatment was tested precisely with cancerous and non-cancerous cells from the same tissue, the same cell type, and culture in the same medium. The concept that H_2_O_2_ is always harmful has been widely revised because it may be therapeutically useful by killing cancer cells selectively. Nevertheless, further research is required to better comprehend the molecular mechanisms involved in cancer and non-cancerous cells and to discover specific NTP operating conditions to enhance selectivity.

Pancreatic ductal adenocarcinoma (PDAC) is a challenging neoplastic disease due to its resistance to radio- and chemotherapy. NTP has shown the potential to eliminate cancer cells through oxidative damage, but its effect on pancreatic stellate cells (PSCs), key players in the invasion and metastasis of PDAC, is not well understood. The study by Privat-Maldonado et al. aimed to investigate the effect of an anti-PDAC NTP treatment on PSCs using mono- and co-cultures of RLT-PSC and Mia PaCa-2 cells, measuring tissue reduction and mRNA expression of PSC activation markers and extracellular matrix remodeling factors [8]. NTP inhibited growth in Mia PaCa-2 cells and co-cultured tissue, but its effectiveness was reduced in the latter. NTP did not alter the mRNA expression of PSC activation and extracellular matrix remodeling markers, and no changes were observed in MMP2 and MMP9 expression in RLT-PSCs, but small changes were observed in Mia PaCa-2 cells. The study concludes that NTP can eliminate PDAC cells without altering PSC growth and phenotype, which is important as PSC elimination is detrimental to PDAC tissue. However, NTP may lead to increased MMP2 and MMP9 expression in Mia PaCa-2 cells from co-cultured tissue, which should be investigated further to determine the effect of NTP on EMT in Mia PaCa-2 cells.

Also, Perrotti et al. contributed to the systematic review focusing on the use of NTP for head and neck cancer (HNC) treatment, which is responsible for many patient deaths due to late diagnosis, recurrence, metastases, and treatment failure [9]. The review summarizes the characteristics and settings of the different NTP devices, in vitro and in vivo treatment protocols, molecular mechanisms of action, and the successes and pitfalls of current NTP applications in HNC. The study includes 24 studies, and most studies use a plasma jet device and argon as the mostly employed working gas. Direct and indirect plasma applications were reported in most studies, and in vitro, investigations were the majority. The review highlights the promising effects of NTP as supporting treatment in HNC, and the need for standardization of current protocols to enable effective clinical translation. Further research is needed to explore the optimal parameters for cancer cell treatment and to use plasma as an alternative means of effective cancer therapy.

### 2.5. Plasma-Based Fungal Cellulose Production

The study by Yu et al. explores the potential of using a non-thermal atmospheric pressure plasma jet to improve the production efficiency of cellulolytic enzymes in Neurospora crassa, a filamentous fungus, for industrial-scale production [10]. The study found that plasma treatment can promote the transcription and secretion of cellulolytic enzymes in the presence of Avicel (induction condition), a cellulose product, by enhancing the intracellular level of NO and Ca^2+.^ The study also found that the total activity of cellulolytic enzymes and protein concentration were significantly increased after 2 and 5 *min* of plasma treatment. The increased levels of Ca^2+^ and intracellular NO may have acted as signals for activating enzyme expression and secretion. The findings suggest that plasma treatment could represent a potential tool for improving enzyme secretion and/or enzyme gene expression, making fungi more reliable producers of industrial enzymes. Further research is needed to fine-tune the plasma treatment conditions for obtaining maximum efficiency of enzyme production and to understand the detailed mechanism(s) underlying plasma-mediated enhancement.

### 2.6. Effect on Lipid Bilayer 

As we already know that NTP is a potential tool for biomedical applications due to its ability to produce RONS for oxidative stress-based therapy. However, the impact of plasma-derived RONS on cell membrane lipids and properties is not fully understood. In a recent study by Nasri et al., the changes in lipid bilayer functionality under oxidative stress generated by an argon plasma jet (kINPen) were investigated [11]. Various asymmetric bilayers mimicking the structure and properties of the erythrocyte cell membrane were transferred onto a gold electrode surface. The study found that cholesterol played an important role in protecting the lipid bilayers against oxidative events and subsequent losses in barrier function. The head group characteristics such as charge, size, and hydrogen bonding capacity also affected the resilience and barrier properties of the lipid layers during NTP treatment. Phosphatidylcholine-based lipid bilayer was most sensitive, while phosphatidylethanolamine, phosphatidylserine, and sphingomyelin-based lipid layers were more robust. Future studies will include the analysis of oxidative stress’s impact on the functionality of membrane proteins.

### 2.7. Plasma-Based Agriculture 

Plasma treatment of seeds could be an eco-friendly and sustainable method to enhance plant growth parameters. The research by Waskow et al. used RNA sequencing to analyze changes in gene transcription in *Arabidopsis thaliana* (L.) Heynh. seeds six days after exposure to surface dielectric barrier discharge plasma treatment [12]. The results showed that plasma treatment time is a parameter that can activate different pathways in plant defense. An 80-*s* treatment upregulated the glucosinolate pathway, which acts as a defense response to insects and herbivores to deter feeding, while a shorter treatment of 60 *s* upregulated the phenylpropanoid pathway, which reinforces the cell wall with lignin and produces antimicrobial compounds, a defense response to bacterial or fungal plant pathogens. The study suggests that plasma treatment can potentially be used in agriculture to protect plants against abiotic and biotic stresses without discharging residues into the environment. However, it should be noted that plasma treatment elicits a wound response in the plant, which could lead to a trade-off between growth and stress/disease resistance, and further studies are needed to explore the possibility of a gene signature profile specific to plasma and to test the survival of plasma-treated plants under realistic conditions. 

### 2.8. Plasma-Induced Osteogenic Differentiation 

NTP has potential in regenerative medicine and orthopedic surgery for bone regeneration. This study by Fischer et al. aimed to determine the biocompatible doses of NTP that would be safe and effective in promoting osteogenic differentiation and immunomodulatory potential in human primary bone marrow mesenchymal stromal cells (hBM-MSCs) [13]. The study used a clinically approved plasma jet and evaluated the effects of NTP exposure on hBM-MSCs from arthroplasty patient cohorts. The researchers found that biocompatible doses of NTP did not enhance hBM-MSCs’ osteogenic capacity but instead led to modest but significant augmentation of anti-inflammatory and proangiogenic cytokines and growth factors. The study provides preclinical data for the potential therapeutic use of NTP in musculoskeletal regeneration, but further evaluation of NTP safety and efficacy in vivo or multicellular in vitro models is necessary.
